# Female migrants, family members and community socio-demographic characteristics influence facility delivery in Rufiji, Tanzania

**DOI:** 10.1186/1471-2393-14-329

**Published:** 2014-09-23

**Authors:** Francis Levira, Lauren Gaydosh, Astha Ramaiya

**Affiliations:** Data Analysis Cluster Unit, Ifakara Health Institute, Plot 463, Kiko Avenue Mikocheni, PO Box 78373, Dar es Salaam, Tanzania; Department of Sociology, Office of Population Research, Princeton University, Princeton, NJ USA; Department of Community Health and Prevention, School of Public Health, Drexel University, Philadelphia, PA USA

**Keywords:** Facility delivery, Community, Determinants, Tanzania, Pregnancy, Place of birth

## Abstract

**Background:**

Health professionals and public health experts in maternal and newborn health encourage women to deliver at health facilities in an effort to reduce maternal and newborn mortality. In the existing literature, there is scant information on how migration, family members and community influence facility delivery. This study addresses this knowledge gap using 10 years of longitudinal surveillance data from a rural district of Tanzania.

**Methods:**

Multilevel logistic regression was used to quantify the influence of hypothesized migration, family and community-level factors on facility delivery while adjusting for known confounders identified in the literature. We report adjusted odds ratios (AOR).

**Results:**

Overall, there has been an increase of 14% in facility delivery over the ten years, from 63% in 2001 to 77% in 2010 (p < .001). Women residing in households with female migrants from outside their community were more likely to give birth in a facility AOR = 1.2 (95% CI 1.11-1.29). Furthermore, the previous facility delivery of sisters and sisters-in-law has a significant influence on women’s facility delivery; AOR = 1.29, 95% CI 1.15-1.45 and AOR = 1.7, 95% CI 1.35-2.13 respectively. Community level characteristics play a role as well; women in communities with higher socioeconomic status and older women of reproductive age had increased odds of facility delivery; AOR = 2.37, 95% CI 1.88-2.98 and AOR = 1.17, 95% CI 1.03-1.32 respectively.

**Conclusion:**

Although there has been an increase in facility delivery over the last decade in Rufiji, this study underscores the importance of female migrants, family members and community in influencing women’s place of delivery. The findings of this study suggest that future interventions designed to increase facility delivery must integrate person-to-person facility delivery promotion, especially through women of the community and within families. Furthermore, the results suggest that investment in formal education of the community and increased community socio-economic status may increase facility delivery.

**Electronic supplementary material:**

The online version of this article (doi:10.1186/1471-2393-14-329) contains supplementary material, which is available to authorized users.

## Background

Health facility delivery plays an important role in improving maternal and newborn health worldwide. Health professionals and public health experts recommend that women deliver at health facilities due to the availability of skilled birth attendants, a clean environment, emergency obstetric services in case of complications, and affordable lifesaving technologies [1, 2]. Yet in Tanzania, only 50% of women deliver in a facility [1].

The determinants of facility delivery can be organized into three categories: individual, institutional and community. First, in Tanzania and other resource poor countries, individual demographic characteristics of the mother are associated with increased facility delivery. Mothers who are less than 20 years or older than 30 years are more likely to deliver at a facility, as well as mothers at low (1–2 births) or high parity (>5 births) [3–9]. Furthermore, unmarried and more educated women are also more likely to give birth in a facility [3–9]. In addition, women’s attitudes towards use and availability of medical services influence their likelihood of delivering in a facility, including subscription to community health insurance, attitudes toward medical doctors, acceptability of traditional birth attendants, attitudes toward facility health delivery and frequency of antenatal care (ANC) visits [4, 6, 8–11]. Moreover, residence in urban areas, higher socioeconomic status and planned pregnancy are associated with greater likelihood of facility delivery [5, 6, 8, 9, 12]. Lack of understanding about the importance of facility delivery and women’s lack of household decision making power have been reported as barriers to facility delivery in other studies [3, 8, 11].

Second, in addition to individual level factors, institutional characteristics of the health facilities where women would attend for delivery have a significant impact on current and future use. The presence of a skilled attendant at a facility has been shown to reduce maternal mortality and morbidity compared to unskilled birth delivery in some developing country settings [13–16]. However, inadequate medical supplies and lack of staff are a major barrier to facility delivery in Tanzania and other resource constrained settings [10, 17, 18]. Unappealing labor environment [10, 18] and abuse or disrespect shown by birth attendants significantly contribute to home deliveries [17, 19]. Accessibility of the facility is another major barrier, where distance and high transport costs have been commonly cited as reasons for delivering at home [10, 16, 18].

Third, whereas individual and health facility factors affecting facility delivery are well-established in the literature, research on community level determinants is limited. One study conducted in six African countries including Tanzania examined community-level factors associated with the decision to deliver in health facilities using the Demographic and Health Surveys (DHS) [5]. In the Tanzania DHS, community factors that increased facility delivery included lower community fertility, greater male approval of family planning, and community-level rates of previous health facility births [5].

Although some studies have tried to incorporate community-level factors, the evidence is limited. Our study contributes to this literature by quantifying the previously unexamined influence of in-migrants, sisters, sisters-in-law and community factors on facility-based delivery in rural Tanzania.

We examine the influence of migrants and family member on women’s likelihood of facility delivery in two ways. First, we estimate the odds of facility delivery in households with female migrants who have moved in from outside the study site. Second, we examine the influence of having sisters or sisters-in-law who previously delivered in health facilities. In this study, sisters and sisters-in-law are referred to as family members.

Literature from Guatemala showed the co-residence of in-migrants was positively associated with women’s contraceptive knowledge and utilization [20]. Another study looking at female migration in African cities found that female migrants had lower fertility rates due to their unmarried status, modern contraceptive usage after two years in the cities, spousal separation in women who were married and extended post-migration abstinence [21]. Due to their migration experience, in-migrants from cities may be more educated about the benefits of facility delivery or more knowledgeable about health services in general. Therefore, we hypothesize that migrant household members may have a positive influence on facility delivery. Taking into consideration the fact that a substantial proportion of migration in our setting occurs between the study area and Dar es Salaam, the biggest city in the country, we hypothesize that migrant household members may have a positive influence on facility delivery.

We examine the influence of family members by considering sisters’ and sisters’-in-law experience with facility delivery. Prior research has shown that village communities and family members play an integral role in shaping individual behavior. Village communities are close knit and strongly enforce social norms [7, 22]. A study in Nepal demonstrated the positive effect of mother-in-law’s ANC utilization on women’s ANC utilization [23]. Furthermore, a study in Kenya indicated that mother and mother-in-law support and advice to deliver at a facility increased facility delivery [7]. Drawing from these findings, we employ a measure of sister’s and sister-in-law’s previous place of delivery, reasoning that family members influence individual women to make birth choices based on their own experience. As such, we hypothesize that the maternal healthcare experience of individuals in a woman’s family is likely to influence her decision of place of delivery.

In addition to these two new variables, we also examine the influence of three community level factors: 1) average educational attainment for women in the community, 2) average household wealth in the community derived from an asset index, and 3) average age of women of reproductive age in the community. We hypothesize that women living in communities with greater levels of female education will be more likely to deliver in the facility due to the combined effect of education, community accessibility to public health knowledge, and autonomy to seek health services. We hypothesize that women in communities of greater wealth will be more likely to deliver in facilities because of their greater ease of accessing health care services. Furthermore, we predict, that women in communities with an older mean age of women of reproductive age will be more likely to deliver in the facility as a result of their greater collective experience of the benefits of skilled birth attendants.

Finally, this paper also tests the hypothesis that, in addition to the observed influence of migration, family member and community factors, there are residual unmeasured community and maternal factors that influence a woman’s decision to give birth in a facility.

## Methods

### Study setting

This study utilizes delivery records of women who gave birth from 2001 to 2010 in the Rufiji Health and Demographic Surveillance System (RHDSS). The surveillance area was established in 1998 with an initial census conducted by enumerating the entire population. The surveillance system covers an area of 1,813 square kilometers with 33 communities (villages). The surveillance area, and lies about 178 km south of Dar es Salaam (the largest city in the country), and covers approximately 50% of the population of Rufiji District. The study area is geographically formed by 33 administrative villages which will be referred to as communities and serve as a unit of analysis in this study. Dominant ethnic groups are Ndengereko, Makonde and Sukuma. The surveillance system is managed by Ifakara Health Institute (IHI).

Rufiji district was the site of two projects aimed at increasing facility delivery. From 1998 – 2003, the Ministry of Health implemented the Tanzania Essential Health Intervention Project (TEHIP). The goal of TEHIP was to increase district capacity to provide expectant mothers with access to basic obstetric and reproductive care. The project provided management tools such as training in management skills, team building, planning, and introduced management systems to help the district focus resources on areas in greatest need [24]. Following TEHIP, the Empower project (2006 – present) upgraded facility infrastructure and supplied equipment to increase access to comprehensive emergency obstetric care. It has trained staff to improve their service provision and increase retention.

### Data

The population under surveillance in Rufiji is a dynamic cohort that enrolls individuals through birth and in-migration and who exit through death or out-migration. Each enumerated individual is given a unique identification number that is used to longitudinally track demographic events. Field teams visit each registered household three times annually to record dates of births, deaths, in and out -migrations and marital status changes that have occurred to each individual since the previous visit. Birth registration includes detailed information about mother’s place of delivery and assistance during delivery. More details about the surveillance system can be found elsewhere [25].

This paper uses data on all deliveries to women aged 15–49 between 2001 and 2010. A total of 20,049 deliveries were included in the analysis. Although the RHDSS started in 1998, data collection on place of delivery began in 2001. The dichotomous outcome variable compares delivery in a facility to delivery at home. The annual percent of facility deliveries was calculated by the ratio of facility deliveries with all deliveries recorded in the surveillance area. The change in the percent of facility delivery was calculated as difference in proportion in 2001 and 2010 at 5% significance level.

The predictor variables of interest were constructed as follows. Presence of an in-migrant in a household was captured from the dataset documenting in and out-migration in the surveillance area. We construct a binary measure indicating presence of an adult female in-migrant in a household two years prior to delivery. Sisters’ place of delivery was constructed by first identifying sisters through matching parent identification numbers, and then determining place of delivery for sisters’ previous births using the birth registry data. We then construct a count variable for how many times she has given birth in a facility. The measure of sisters-in-law’s place of delivery is similarly constructed; women are linked to their husbands, and we then use husband’s identification number to link him to his sisters through matching parent identification numbers. We included sisters-in-law’s experience with facility delivery because Rufiji is a patrilineal society, and women are likely to reside in their husbands’ home village. To capture the influence of the community, we calculate the mean age of women of reproductive age, mean household wealth, and mean years of education of women of reproductive age by village using all residents in the village at the time of birth.

Control measures were constructed as follows. Wealth measures were constructed using an asset index constructed through principal components analysis, based on asset ownership (such as bicycle, radio, bed) and structural characteristics of the home (such as roofing and building material) [26]. Asset data are collected annually. Maternal characteristics of education, work (paid/other) and marital status were captured at baseline or during routine updates of household data. Parity, multiple births, season of birth, place of delivery and outcome of every pregnancy were extracted from birth registration data. Distance to the nearest health facility was calculated from GPS points mapped at each household and health facility in the surveillance area.

Mapping of geographic boundaries was conducted to locate all 33 communities and main roads. Proportion of facility delivery for each community was added to the surveillance map to geographically explore the facility delivery in relation to village main road connections.

### Model specification

Exploratory analysis was conducted by plotting annual proportions of facility delivery (number of reproductive aged women who delivered at the facility/total number of deliveries) by socio-economic status, education, previous birth outcome and previous place of delivery.

Multilevel logistic regression was used to estimate the hypothesized influence of in-migrants, family and community-level factors on facility delivery while adjusting for known confounders including mother’s age, birth order, season of birth, place of delivery of previous pregnancy, and marital status. Multilevel models were specified where children are nested within their mothers and mothers within communities. Mother and community level random effects were included because many individual and community-level factors that may influence facility delivery are not collected or are unobserved, such as husband’s approval of facility delivery and tribe. Maximum likelihood was used to estimate regression model coefficients and results are presented as odds ratios with 95% confidence interval.

### Ethics

The study protocol was approved by Ifakara Health Institution Internal Review Board, National Institute of Health Research and Commission of Science and Technology, Tanzania. All participants were explained the demographic surveillance system study protocol and were enrolled through informed consent.

Our research had adhered to the STROBE guidelines for observational cohort studies (Additional file 1).

## Results

**Figure 1:**
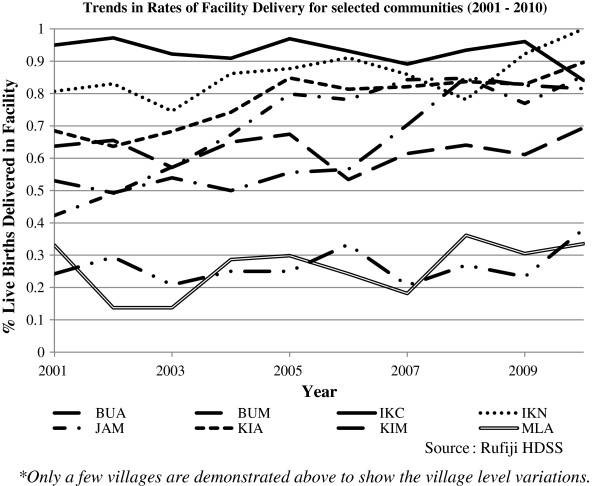
**Trends in community rates of facility delivery in RHDSS, 2001 –2010.**

Overall, there has been an increase of 14 percentage points in facility delivery over the ten years, from 63% in 2001 to 77% in 2010 (p < .001). However, this increase has not been uniform across communities. Over the study period there has been substantial variation in the trend and proportion of facility based deliveries across communities. In Figure 1 we show a few of the communities to demonstrate differences in trajectories of facility delivery. Communities such as Ikwiriri Central (IKC) and Ikwiriri North (IKN) have a consistently high rate of 95% throughout the 10 years. In other communities, such as Jaribu Mpakani (JAM), facility deliveries increased considerably, from 40% to 85%. However, in two of the communities (Mlanzi (MLA) and Bumba/Msoro (BUM), the facility delivery rate is consistently low, between 30% to 40%.

**Figure 2:**
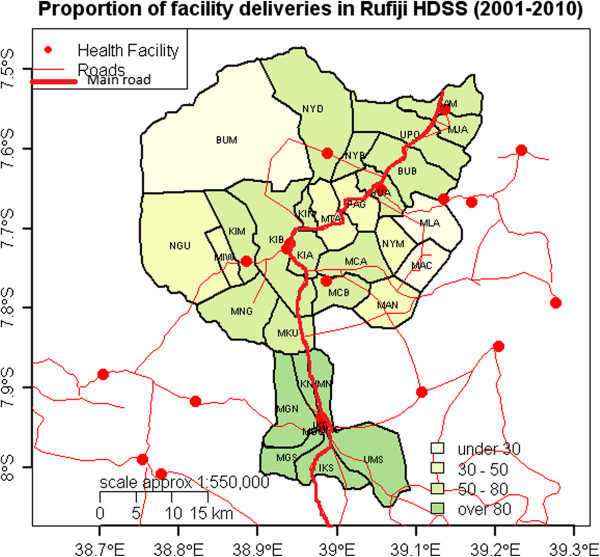
**Proportion of facility deliveries in RHDSS, 2001 – 2010.**

To explore potential reasons for this variation, Figure 2 shows the average proportion of facility delivery in all 33 communities over 10 years. Three main features that could explain geographic variations are remoteness of the communities, health facility availability and main road accessibility. Remote communities of Ngulakula (NGU), BUM, Nyamwimbe (NYM), Machepe (MAC), MLA and Mangwi (MAN) have a facility delivery rate of less than 50% over the study period. These communities do not have access to a main road. Communities located in large centers of Ikwiriri, Kibiti and Mchukwi are accessible to 3 health centers, 1 large health center and a hospital respectively. These exploratory findings suggest that geographic characteristics of the village may influence facility delivery.

**Figure 3:**
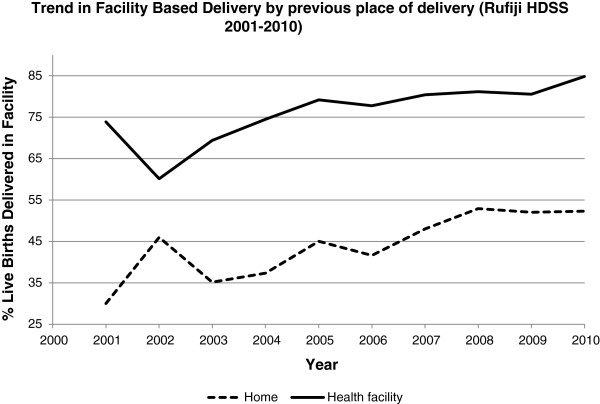
**Facility based delivery differential in RHDSS, 2001–2010.**

Figure 3 shows results of a crude analysis including a few variables that may account for differential facility delivery. The proportion of women delivering in a facility is consistently higher for women in the highest wealth quintile compared to women in the lowest wealth quintile. Similarly, women with at least a primary school education have consistently higher rates of facility delivery than women with no education. Previous birth outcome appears important as well, with women who experienced an abortion/miscarriage/stillbirth in the previous pregnancy having higher rates of facility delivery than women who had a live birth. Finally, women with previous experience with facility delivery have higher rates of facility delivery than those who previously delivered at home.

In Table 1 we present results for an individual level model and a model with individual and community level factors. For Model 1, we estimate a simple logistic regression excluding community level variables and random effects for the mother and village. The odds of facility delivery increased by 31% for women in households with female in-migrants (Adjusted odds ratio (AOR) =1.31, 95% CI: 1.23-1.4). For Model 2, we add community level factors and random effects for mother and community. Consistent with the results from Model 1, the odds of facility delivery increased by 20% for women with female in-migrants in the household (AOR = 1.20, 95% CI 1.11-1.29).

**Table 1:** **Logistic regression analysis on the determinants of facility delivery in Rufiji-Model: 1-2**

	Model 1*	Model 2**
	n = 19,992	n = 19,992
	OR (95% CI)	OR (95% CI)
**Individual level factors**		
Mother’s age	0.96(0.93-0.99)	0.93(0.9-0.97)
Mother’s age^2^	1(1)	1(1)
Parity	0.89(0.87-0.91)	0.89(0.87-0.92)
Education level		
*No education*	ref	ref
*Primary education*	1.03(0.96-1.1)	1.16(1.07-1.25)
*Secondary +*	1.48(1.16-1.88)	1.75(1.35-2.27)
Female migrants		
*No female migrants*	ref	ref
*Female migrants*	1.31(1.23-1.4)	1.2(1.11-1.29)
Occupation		
*Famers*	ref	ref
*Paid work*	1.72(1.55-1.91)	1.56(1.39-1.76)
*Other work*	1.61(1.4-1.84)	1.53(1.31-1.79)
Distance from facility	0.93(0.92-0.94)	0.98(0.97-0.99)
Multiple birth		
*Single*	ref	ref
*Twins*	1.6(1.32-1.94)	1.5(1.22-1.84)
Marital status		
*Single*	ref	ref
*Married*	0.99(0.92-1.06)	1.01(0.93-1.1)
Season		
*Dry*	ref	ref
*Raining*	0.89(0.83-0.95)	0.9(0.84-0.97)
Economic status		
*Poorest*	ref	ref
*Quintile 2*	1.21(1.1-1.33)	1.13(1.02-1.26)
*Quintile 3*	1.45(1.32-1.59)	1.32(1.19-1.47)
*Quintile 4*	1.96(1.77-2.17)	1.59(1.42-1.78)
*Least poor*	3.55(3.12-4.04)	2.66(2.3-3.07)
Previous place of delivery		
*Health facility*	ref	ref
*Not known/First birth*	0.38(0.34-0.42)	0.41(0.36-0.46)
*Home*	0.32(0.29-0.35)	0.51(0.45-0.56)
Previous pregnancy outcome		
*Live birth*	ref	ref
*Not known/First birth*	1.68(1.51-1.87)	1.77(1.57-2.01)
*Still Birth/Miscarriages/Abortion*	1.87(1.41-2.48)	1.92(1.42-2.6)
**Community – level factors**		
Female community years of schooling		0.72(0.52-0.99)
Community asset index		2.37(1.88-2.98)
Female mean age of community		1.17(1.03-1.32)
**Random effects**		
Region [SE]		0.58(0.44-0 .75)
Mother [SE]		0.52(0.42-0.65)

Ref = Reference category/comparison group.

SE = Standard error of random variance.

*Simple logistic regression with individual level variable.

**Multilevel logistic regression model with individual and community level variables and random effects variables for mother and village.

Community level factors identified in Model 2 were high community asset index (AOR = 2.37, 95% CI 1.88-2.98), and high average age of females in the community (AOR = 1.17, 95% CI 1.03-1.32). Increased female education in the community was weakly associated with decreased odds of family delivery (AOR = 0.72, 95% CI 0.52-0.99).

Models 1 and 2 identified individual level factors associated with facility delivery that are well supported and established in the literature. This includes younger age of women, lower parity, higher education (primary and above), high income job (compared to agriculture), high economic status, multiple birth, dry season, previous health facility delivery and previous poor birth outcome (stillbirth/miscarriage/abortion). The random effect is significant for the community but insignificant for the mother, suggesting that there are unobserved community characteristics that influence facility delivery

In Table 2 we test the influence of sisters’ (Model 3) and sisters-in-laws’ facility delivery experience (Model 4). Models 3 and 4 are separated from Models 1 and 2 due to a large number of missing information on sisters’ variables. The sample size in Models 3 and 4 are reduced to 2240 and 909 deliveries, respectively. This is due to an inability to link sisters, as well as a lack of sisters who gave birth in the interval.

**Table 2:** **Logistic regression analysis on the determinants of facility delivery in Rufiji-Model: 3-4**

	Model 3*	Model 4**
	(n = 2240)	n = 910
	OR (95% CI)	OR (95% CI)
**Individual level factors**		
Mother’s age	0.84(0.74-0.96)	1.03(0.84-1.25)
Mother’s age^2^	1(1–1.01)	1(1)
Parity	0.87(0.77-0.97)	0.83(0.7-0.98)
Education level		
*No education*	ref	ref
*Primary education*	1.22(0.97-1.55)	0.98(0.68-1.4)
*Secondary +*	1.61(0.86-3.03)	0.41(0.13-1.29)
Female migrants		
*No female migrants*	ref	ref
*Female migrants*	1.2(0.96-1.5)	1.28(0.9-1.83)
Occupation		
Famers	ref	ref
*Paid work*	1.41(1.05-1.89)	1.44(0.84-2.49)
*Other work*	1.12(0.8-1.58)	2.6(0.88-7.71)
Distance from facility	0.97(0.94-1)	0.94(0.9-0.99)
Multiple birth		
*No*	ref	ref
*Yes*	1.37(0.72-2.6)	0.46(0.17-1.21)
Marital status		
*Not currently married*	ref	ref
*Married*	1(0.78-1.27)	1.05(0.68-1.6)
Season		
*Dry*	ref	ref
*Raining*	0.92(0.73-1.15)	0.85(0.6-1.23)
Economic status		
*Poorest*	ref	ref
*Quintile 2*	1.39(0.99-1.93)	1.63(0.95-2.8)
*Quintile 3*	1.41(1.02-1.96)	1.08(0.65-1.79)
*Quintile 4*	1.51(1.07-2.13)	1.5(0.88-2.57)
*Least poor*	2.09(1.37-3.19)	2.97(1.41-6.27)
Previous place of delivery		
*Health facility*	ref	ref
*Not known/First birth*	0.67(0.43-1.03)	0.99(0.45-2.18)
*Home*	0.57(0.4-0.79)	0.53(0.34-0.83)
Previous pregnancy outcome		
*Live birth*	ref	ref
*Not known/First birth*	1.43(0.91-2.25)	0.9(0.4-2.06)
*Still Birth/Miscarriages/Abortion*	0.7(0.26-1.86)	5.59(1.11-28.23)
Sister’s previous facility delivery		
*Home*	ref	ref
*Health facility*	1.29(1.15-1.45)	
Number of previous sisters-in-law facility delivery		1.7(1.35-2.13)
**Community – level factors**		
Female community years of schooling	0.78(0.53-1.16)	0.67(0.46-0.97)
Community asset index	1.93(1.45-2.57)	2.13(1.51-3.01)
Female mean age of community	1.17(0.99-1.39)	1.28(1.09-1.52)
**Random effects**		
Region [SE]	0.55(0.38-0.80)	0.34(0.15-0 .79)
Mother [SE]	0.35(0.09-1.37)	0.28(0–8.9)

Ref = Reference category/comparison group.

SE = Standard Error of random variance.

*Multilevel logistic regression model with random effects for mother and village with sister variable.

**Multilevel logistic regression model with random effects for mother and village with sisters and sister-in-law variables.

In Model 3, odds of delivering in a facility significantly increased by 29% (AOR = 1.29, 95% CI: 1.15-1.45) for every increase in number of facility deliveries experienced by her sister. Model 4 shows the odds of facility delivery significantly increased for a unit increase in number of sister’s-in-law facility delivery (AOR = 1.70, 95% CI: 1.35-2.13).

With respect to average community wealth, in Model 3 we find that, women in communities with higher socio-economic status have increased odds of facility delivery (AOR = 1.93, 95% CI: 1.45 – 2.57). Results are consistent in Model 4, (AOR = 2.13, 95% CI: 1.51-3.01).

In Model 4 we also find that higher mean age of women in the community is positively associated with the odds of facility delivery (AOR = 1.28, 95% CI: 1.09 – 1.52). Similar to Model 2, Model 3 also shows a negative influence of community levels of female education (AOR = 0.67, 95% CI 0.46-0.97). Despite controlling for individual and community level variables, the random effect for the community is still significant, which suggests that there are unobserved characteristics that influence facility delivery.

## Discussion

Facility delivery in the RHDSS has been consistently above 70% since 2007, well above the national average of 50% [1]. An overall increase in facility delivery has been observed over the last decade. However, the crude analysis obscures community level variation that indicates a few communities increasing uptake, while most remained stable (high or low) throughout the last decade. Although the government has implemented several supply side programs, the influence of migrants, family and community members on women’s likelihood of delivering at a facility has not been fully researched. Results from this study demonstrate female migrants into household and sisters’ and sisters-in-law’s prior experience with facility delivery positively influence women’s odds of facility delivery. At the community level, high wealth and high mean ages of women are important determinants of facility delivery. However, unobserved community level characteristics are still significant and need to be further investigated.

The lower odds of facility delivery at older ages observed in this study at individual and community level analysis are inconsistent with findings from previous analysis of DHS surveys in Tanzania [5]. These findings are small and barely significant, with approximately 1-2% decline in the odds of facility delivery for a year increase in maternal age.

Higher levels of female education in the community are associated with lower odds of facility delivery, which is opposite from the association with individual levels of schooling. This may be due to the fact that the majority of women in the surveillance area are either illiterate or have incomplete primary education.

This study finds a positive association between the presence of a female in-migrant in the household and the odds of facility delivery. Female in-migrants who enter households from outside the study area may influence or challenge local knowledge with respect to health seeking behavior, hence changing their attitudes toward facility delivery.

Sisters’ and sisters-in-law’s experience with facility delivery positively influences women’s odds of their own facility delivery, demonstrating the importance of family members in communicating information about the health system. Indeed, family members have been previously shown to be a factor in health facility utilization [27]. However, these results should be interpreted with caution, as the sample size decreased drastically for sister and sister-in-law analyses. Nevertheless, this finding is consistent with evidence from Kenya where mother’s and mother-in-law’s advice to deliver at a facility exerted a positive influence on women’s likelihood of delivering at a facility [7].

Previous studies have assessed community level variables through qualitative and quantitative studies. Our study has quantified the influence of community wealth, level of education and maternal age on facility based delivery.

This study contributes to our knowledge about unexplained community level variation in women’s choice of facility delivery. Some studies have added a random effect for the village/cluster, family and the individuals whereas others have assessed specific factors theorized to influence facility delivery [6, 23, 28–30]. The random effects of the community were significant for facility delivery, immunization and care uptake in Kenya and Guatemala respectively [6, 29, 30], consistent with our findings.

The findings of this study suggest that future interventions designed to increase facility delivery must integrate person-to-person facility delivery promotion, especially through women of the community. Furthermore, the results suggest that investment in formal education of the community and increased community wealth may increase facility delivery.

Although this study attempted to quantify the influence of community level variables without losing power, we are limited by the available data. This is most evident with the stark drop in observations when including sisters and sisters-in-law in the model. Due to missing data on sisters and sisters-in-law for the majority of women, it was difficult to assess these factors with such a small sample size. Although the sample size decreased significantly, the remaining sample was sufficiently large (>900 mothers) due to the long individual follow-up. Furthermore, different settings may have differing determinants of facility delivery, limiting the generalizability of our findings. Nevertheless, because this is a longitudinal study in a remote setting, the results of this study may be applicable to similar settings.

There is a need for further qualitative research within urban and rural areas to determine which factors influence facility delivery. Although the supply side of providing quality reproductive healthcare at facilities is progressing, utilization and demand require a better understanding of the family and community contexts in which women live.

## Conclusions

Overall, there has been an increase in facility delivery over the decade, from 63% in 2001 to 77% in 2010. However, this increase has not been consistent across all communities in Rufiji. This study underscores the importance of female migrants, family members and community level factors in influencing women’s place of delivery. The findings of this study suggest that future interventions designed to increase facility delivery must integrate person-to-person facility delivery promotion, especially through women of the community. Furthermore, the results suggest that investment in formal education of women in the community and increased community wealth may increase facility delivery.

## Electronic supplementary material

Additional file 1: **STROBE checklist for cohort studies.** (DOC 80 KB)

## Abbreviations

95% CI: 95% confidence interval
ANC: Antenatal care
IHI: Ifakara health institute
OR: Odds ratio
RHDSS: Rufiji health and demographic surveillance system
TEHIP: Tanzania essential health intervention project
IKN: Ikwiriri North
IKN: Ikwiriri Central
IKS: Ikwiriri South
UMN: Umwe North
UMC: Umwe Central
UMS: Umwe South
MGN: Mgomba North
MGC: Mgomba Central
MGS: Mgomba South
KIM: Kimbuga
NGU: Ngulakula
KIB: Kibiti B
MTA: Mtawanya
MNG: Mng’aru
MIW: Miwaga
BUM: Bumba/Msoro
KIA: Kibiti A
NYD: Nyambunda
NYB: Nyambili
UPO: Uponda
BUB: Bungu B
BUA: Bungu A
MIA: Mjawa
PAG: Pagai
JAM: Jaribu Mpakani
MLA: Mlanzi
NYM: Nyamwimbe
MAN: Mangwi
MCA: Mchukwi “A”
MCB: Mchukwi “B”
MKU: Mkupuka
RUN: Rungungu
KIL: Kilula Tambwe
NYA: Nyamatanga
RUA: Ruaruke A
RUB: Ruaruke B
KIN: Kinyanya
MAC: Machepe
RAA: Ruaruke A
RAB: Ruaruke B
NYG: Nyamatanga
RUG: Rungungu
KIT: Kilula Tambwe.
